# Central dentinogenic ghost cell tumor of the maxilla: a case report with new imaging findings and review of the literature

**DOI:** 10.1007/s11282-024-00764-4

**Published:** 2024-07-05

**Authors:** Suzuka Yoshida, Yohei Takeshita, Toshiyuki Kawazu, Miki Hisatomi, Shunsuke Okada, Mamiko Fujikura, Kyoichi Obata, Kiyofumi Takabatake, Saori Yoshida, Junichi Asaumi

**Affiliations:** 1 Department of Oral and Maxillofacial Radiology, Okayama University Graduate School of Medicine, Dentistry and Pharmaceutical Sciences, 2-5-1 Shikata-cho, Kita-ku, Okayama, 700-8558 Japan; 2 Department of Oral and Maxillofacial Radiology, Faculty of Medicine, Dentistry and Pharmaceutical Sciences, Okayama University, 2-5-1 Shikata-cho, Kita-ku, Okayama, 700-8558 Japan; 3 Department of Oral and Maxillofacial Radiology, Okayama University Hospital, 2-5-1 Shikata-cho, Kita-ku, Okayama, 700-8558 Japan; 4 Department of Oral and Maxillofacial Surgery, Okayama University Hospital, 2-5-1 Shikata-cho, Kita-ku, Okayama, 700-8558 Japan; 5 Department of Oral Pathology and Medicine, Faculty of Medicine, Dentistry and Pharmaceutical Sciences, Okayama University, 2-5-1 Shikata-cho, Kita-ku, Okayama, 700-8558 Japan; 6 Preliminary Examination Room, Okayama University Hospital, 2-5-1 Shikata-cho, Kita-ku, Okayama, 700-8558 Japan

**Keywords:** Dentinogenic ghost cell tumor, Benign odontogenic tumor, Maxilla, Calcification, Computed tomography, Magnetic resonance imaging

## Abstract

A dentinogenic ghost cell tumor (DGCT) is a rare benign odontogenic tumor that commonly shows characteristics of solid proliferation and has a relatively high risk of recurrence after surgical treatment. We herein report a case of a central DGCT that occurred in the maxilla and resulted in bone expansion. This study highlights new imaging findings (particularly magnetic resonance imaging) along with histopathological observations. In addition, we conducted a review of the existing literature on this rare tumor. A 37-year-old man developed swelling around the right cheek. A benign odontogenic tumor such as ameloblastoma was suspected based on the imaging examination findings (including bone expansion and the internal characteristics of the tumor) on panoramic imaging, computed tomography, and magnetic resonance imaging. The lesion was surgically excised from the right maxilla. Postoperative histopathological examination led to a definitive diagnosis of central DGCT. The tumor comprised epithelial neoplastic islands, resembling ameloblastoma, inside tight fibroconnective tissue; masses of ghost cells and formation of dentin were also observed. We had suspected that the minute high-density region around the molars on the imaging examinations represented alveolar bone change; however, it represented dentin formation. This led to difficulty diagnosing the lesion. Although DGCT may present characteristic findings on imaging examinations, its occurrence is infrequent, and in some cases, the findings may include the presence or absence of an impacted tooth without obvious calcification. The present case suggests that we should consider the possibility of an odontogenic tumor with calcification when high-density structures are observed inside the lesion.

## Introduction

A dentinogenic ghost cell tumor (DGCT) is an extremely uncommon benign odontogenic tumor, and it commonly shows solid proliferation in a central/intraosseous or peripheral/extraosseous location [1, 2]. DGCTs consist of epithelial neoplastic islands resembling ameloblastoma accompanied by ghost cells and dentin [2, 3]. The World Health Organization (WHO) previously classified DGCT as a solid type of calcifying odontogenic cyst (COC). In 2005, COC was renamed calcifying cystic odontogenic tumor (CCOT), and a DGCT showing characteristics of solid proliferation was treated as an independent disease. Both were classified as an odontogenic tumor by the WHO. However, the WHO then reclassified CCOT as an odontogenic cyst and renamed it COC in 2017. By contrast, DGCT has been classified as an odontogenic tumor since 2005. DGCT often shows local infiltration and has a relatively high risk of recurrence after surgical treatment despite its benign nature [2, 4, 5].

We herein present a case of a central DGCT that occurred in the maxilla and resulted in bone expansion. We present new imaging findings (especially magnetic resonance imaging [MRI]) along with histopathological observations, and we review the existing literature on this rare tumor.

## Case report

In November 2021, a 37-year-old man visited a general dental practitioner because of swelling around the right cheek. He underwent gingival puncture aspiration by the dental practitioner, and yellow transparent fluid was removed. At the end of January 2022, the dental practitioner referred him to Okayama University Hospital for further examination because the swelling around the right cheek had not decreased in size. The patient’s medical history included autism spectrum disorder, intellectual disability, type II diabetes mellitus, and hyperlipidemia.

**Fig. 1:**
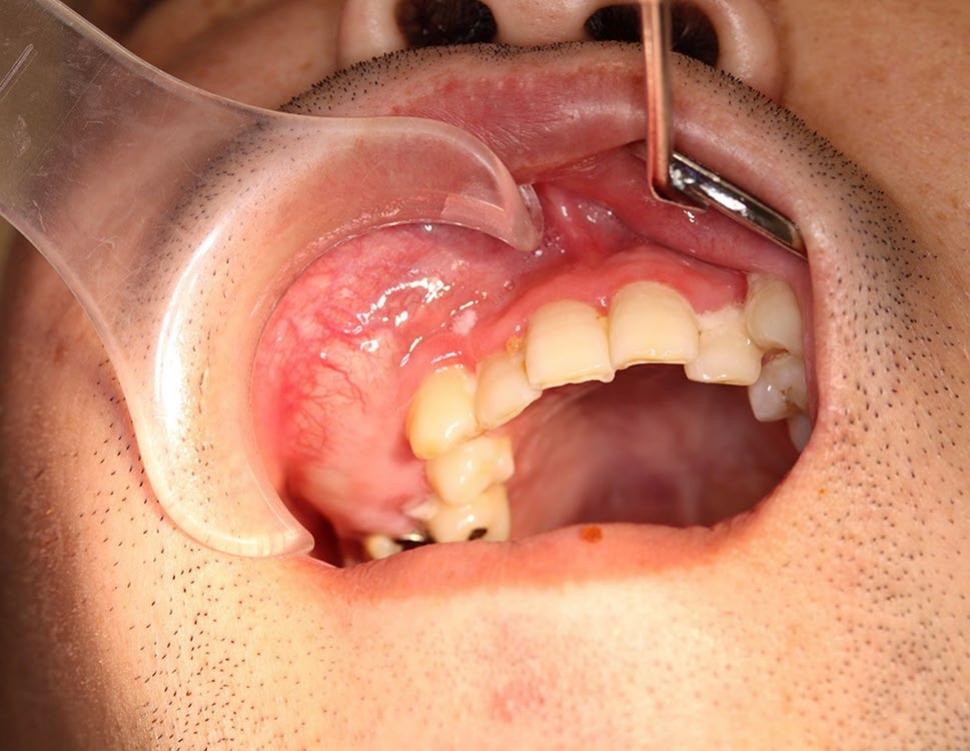
Intraoral findings at the time of the initial examination. Intraoral examination revealed a mass with rippling from the maxillary right anterior teeth to the molars

**Fig. 2:**
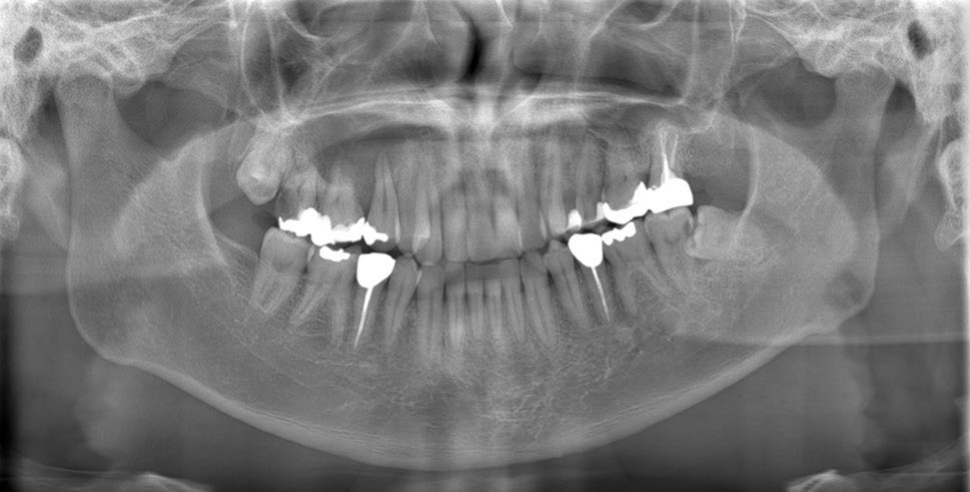
Preoperative panoramic image showing relatively well-defined, homogeneous, and round radiolucent lesion occupying the right maxillary sinus from the root apices of teeth 14 to 17

At the time of the initial clinical examination at Okayama University Hospital, extraoral examination revealed swelling of the right cheek, difficulty in opening the mouth, and swelling of the cervical lymph nodes. Intraoral examination revealed a mass with rippling from the maxillary right anterior teeth to the molars (Fig. 1). A relatively well-defined, homogeneous, and round radiolucent region occupied the right maxillary sinus from the root apices of teeth 14 to 17 on a panoramic image (Fig. 2). The floor line of the right maxillary sinus was suspected to be elevated and was recessed between teeth 15 and 16. The root apices of teeth 15 to 17 were included in the radiolucent region, and root resorption of tooth 16 was suspected. We suspected an odontogenic cyst, such as an odontogenic keratocyst or a benign odontogenic tumor such as ameloblastoma.

**Fig. 3:**
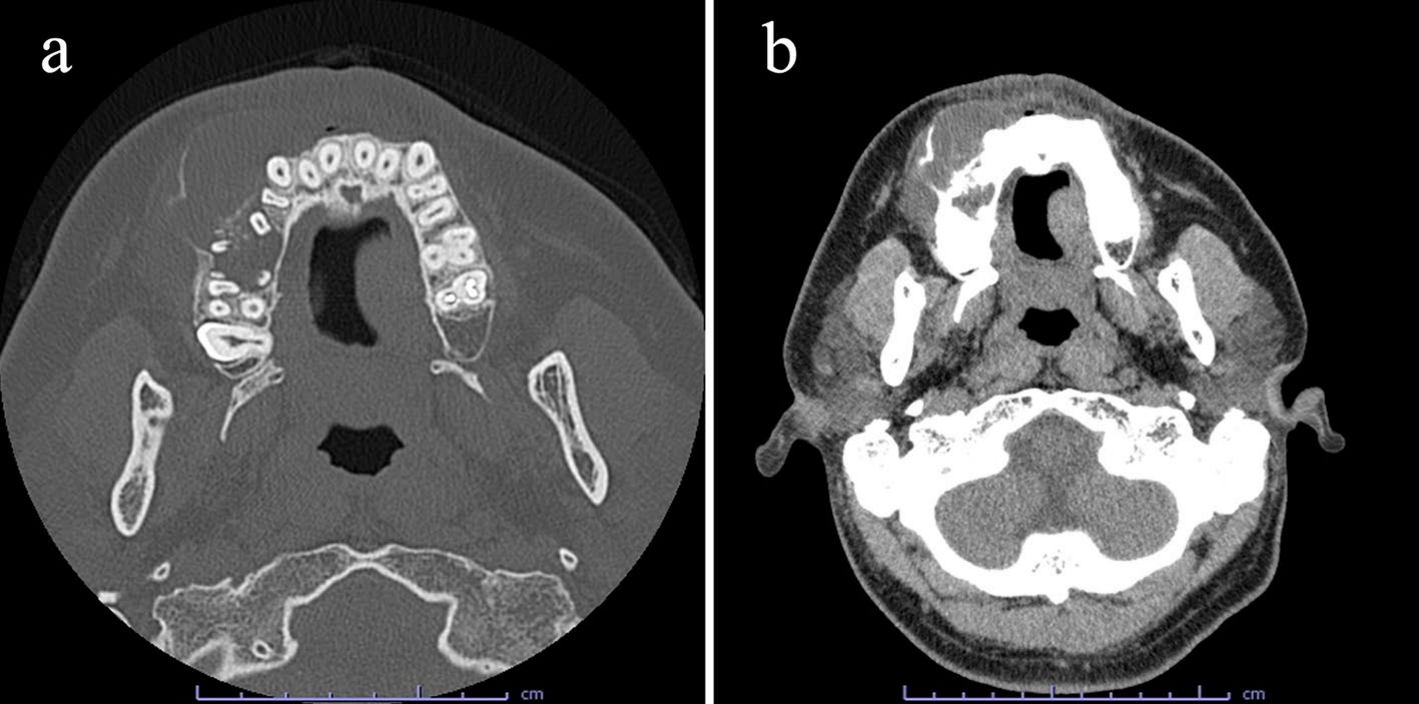
Computed tomography images. **a** Axial image of the bone windows showing a well-defined, multilocular, and low-density region on the right side of the maxilla with marked buccal swelling. The minute high-density region was observed around the root apices of premolar to molar teeth, **b** Axial image of the soft tissue windows showing a homogeneous low-density region

Computed tomography (CT) images showed a well-defined, multilocular, and low-density lesion on the right side of the maxilla with marked buccal swelling on bone windows. The minute high-density region which suspected the alveolar bone appeared to have been coarsely absorbed was observed around the root apices of premolar to molar teeth (Fig. 3a). The lesion also showed homogeneous low density on soft tissue windows (Fig. 3b). Slight root resorption at the root apices of teeth 14, 16, and 17 was observed. There were no obvious inflammatory findings in the surrounding tissues. Based on the degree of bone expansion and internal density, a benign odontogenic tumor such as ameloblastoma was suspected.

**Fig. 4:**
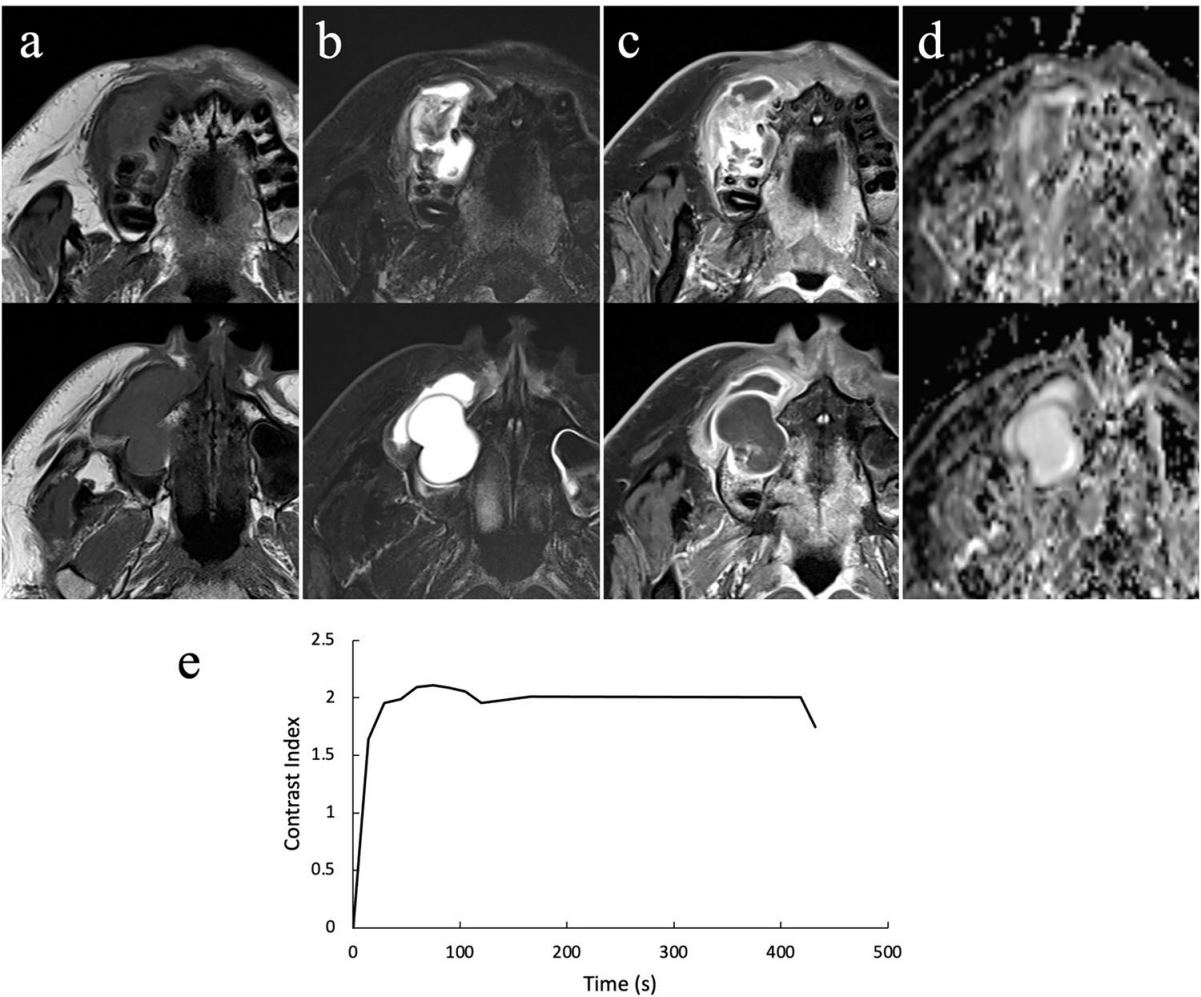
Contrast-enhanced magnetic resonance imaging showing a multilocular region around the right maxilla. **a** Axial T1-weighted image showing homogeneous isointensity, **b** Axial short T1 inversion recovery image showing homogeneous strong hyperintensity affecting almost all of the region, whereas the area around the alveolar region showed heterogeneous hypointensity to hyperintensity, **c** Axial contrast-enhanced T1-weighted image showing heterogeneous enhancement around the alveolar region at the lower site of the lesion and enhancement along the margin at the upper site of the lesion, **d** The apparent diffusion coefficient map showing the value at 1.5 × 10^−3^ mm^2^/s around the alveolar region at the lower site of the lesion, and high at 2.8 × 10^−3^ mm^2^/s at the upper site of the lesion, **e** The contrast index curve rapidly increased and reached a plateau at approximately 30 s, and the plateau was sustained to approximately 400 s

MRI showed a multilocular region around the right maxilla. This region showed homogeneous isointensity on T1-weighted images (T1WI) (Fig. 4a). Almost all of the region showed homogeneous strong hyperintensity that suspected the cystic region, whereas the area around the alveolar region showed heterogeneous hypointensity to hyperintensity that suspected the solid region on short T1 inversion recovery (STIR) images (Fig. 4b). Contrast-enhanced T1WI showed heterogeneous enhancement around the alveolar region at the lower half site of the lesion that suspected the solid region and enhancement along the margin at the upper site of the lesion that suspected the cystic region (Fig. 4c). The apparent diffusion coefficient (ADC) was at 1.5 × 10^−3^ mm^2^/s around the alveolar region at the lower site of the lesion, and high at 2.8 × 10^−3^ mm^2^/s at the upper site of the lesion on ADC map (Fig. 4d). A contrast index (CI) curve was created using the signal intensity (SI) on dynamic contrast-enhanced MRI. The CI was calculated from the formula CI = [SI (post-contrast) − SI (pre-contrast)] / SI (pre-contrast). The CI curve rapidly increased and reached a plateau at approximately 30 s, and the plateau was sustained to approximately 400 s (Fig. 4e). Based on the above findings including heterogeneous enhancement at the lower half site of the lesion, the value of ADC and the pattern of the CI curve, a benign odontogenic tumor such as ameloblastoma was suspected.

**Fig. 5:**
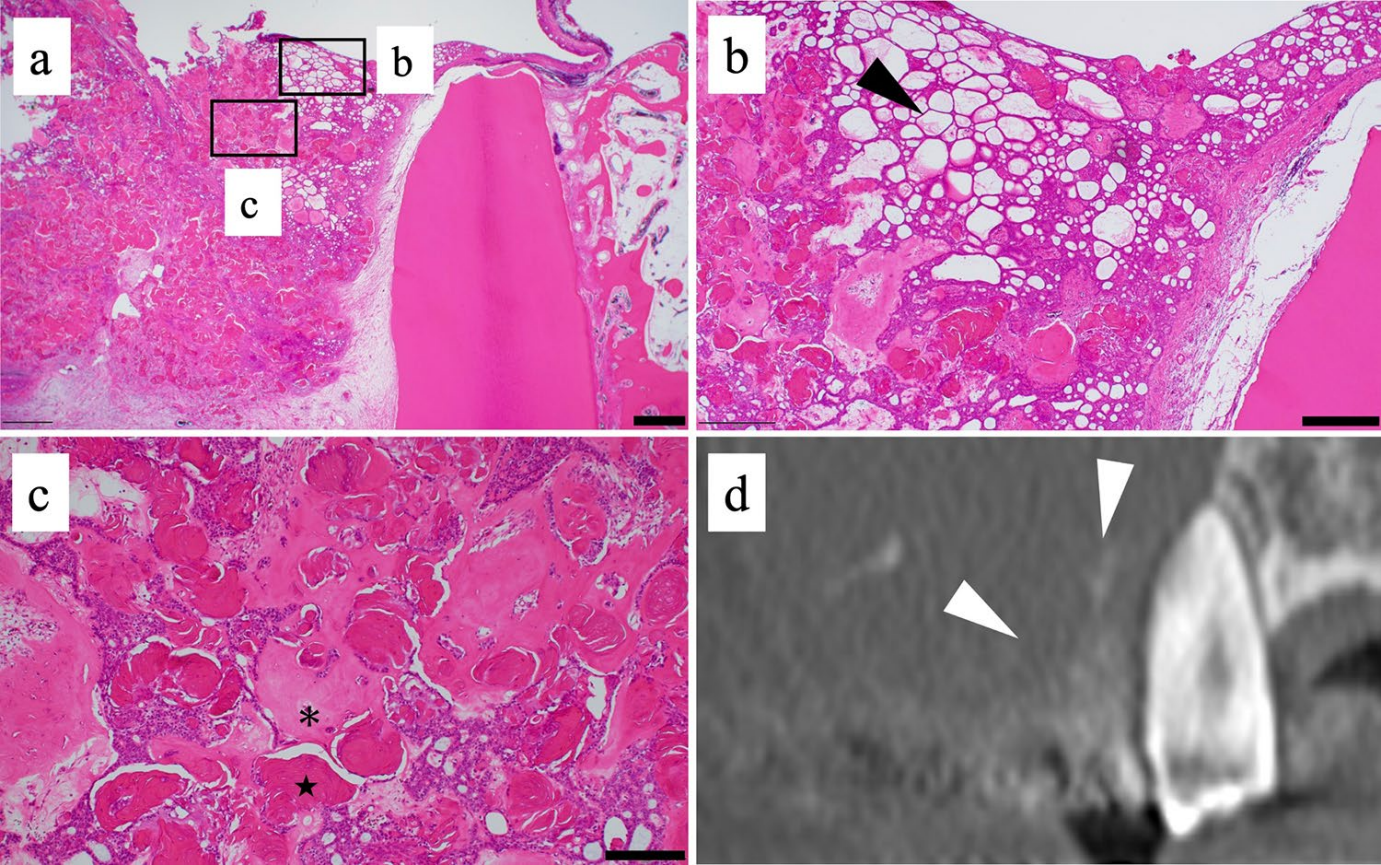
Histopathological findings of the tumor around the right maxilla. **a** The tumor formed a mass around the root apex of tooth 14. Scale bar: 1 mm, **b** The tumor comprised epithelial neoplastic islands resembling ameloblastoma (black arrowhead) inside tight fibroconnective tissue. Scale bar: 500 μm, **c** Masses of ghost cells and formation of dentin were also observed (★ghost cell, *dentin). Scale bar: 200 μm, **d** Coronal computed tomography image of the right maxilla around tooth 14. The region of the alveolar bone that appeared to have been partially coarsely altered (white arrowheads) was found to be fine dentin

**Fig. 6:**
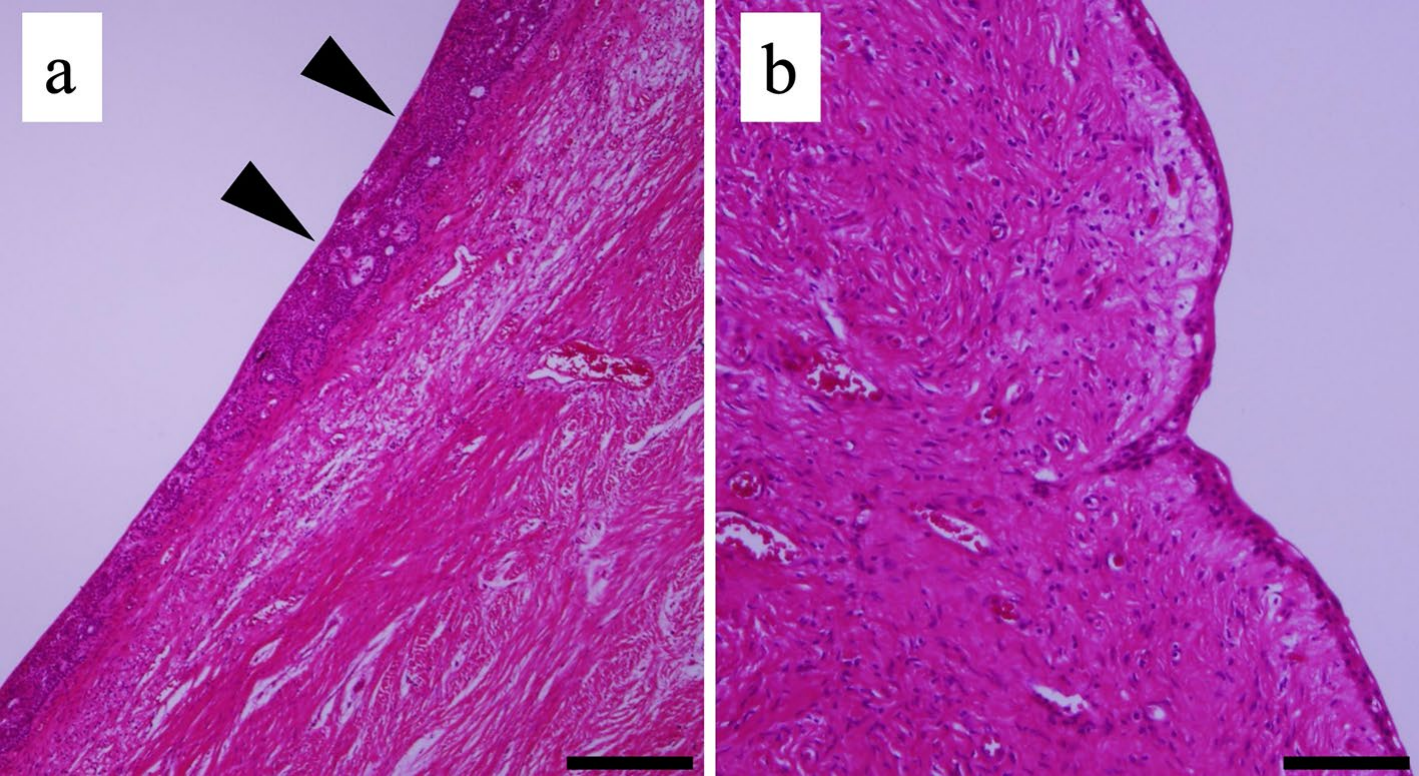
Histopathological findings of the tumor at the boundary between the solid region and cystic region, **a** Epithelial neoplastic islands resembling ameloblastoma (black arrowhead) were observed at the boundary. Scale bar: 1 mm, **b** The epithelium relining the cystic region was thin stratified squamous epithelium similar to that seen in a dentigerous cyst. Scale bar: 1 mm

Right partial maxillectomy was performed under general anesthesia in May 2022. On postoperative histopathological examination, the tumor comprised epithelial neoplastic islands resembling ameloblastoma inside tight fibroconnective tissue (Fig. 5a, b). Masses of ghost cells and formation of dentin were also observed (Fig. 5c). The region of the alveolar bone that appeared to have been partially coarsely altered on CT images was found to be fine dentin when compared with the histopathologic findings (Fig. 5d). No ghost cells were observed at the boundary between the solid region (which showed heterogeneous enhancement around the alveolar region at the lower site of the lesion) and the cystic region (which showed enhancement along the margin at the upper site of the lesion). However, epithelial neoplastic islands resembling ameloblastoma were observed at the boundary (Fig. 6a). The epithelium relining the cystic region was thin stratified squamous epithelium similar to that in a dentigerous cyst (Fig. 6b). Based on the above findings, the final diagnosis was central DGCT.

## Discussion

DGCT accounts for approximately 0.28% to 0.38% of all odontogenic tumors and is thus an extremely uncommon benign odontogenic tumor [1, 2, 6, 7]. DGCT can occur in all age groups but shows higher frequencies in middle-aged to elderly patients [2, 7]. A male predilection has been noted in the literature [1, 2]. DGCT is more commonly found in the mandible than maxilla [2]. More cases of a central/intraosseous pattern than peripheral/extraosseous pattern have been reported [1, 2].

Our patient presented with a mass in the right maxilla but did not complain of pain at the site of origin. Instead, he had difficult in mouth opening and developed swelling of the right buccal region and cervical lymph nodes. We considered that the swelling was the result of bone expansion caused by the mass, and the patient’s history of autism spectrum disorder and intellectual disability might have resulted in his late presentation to the hospital.

DGCT presents certain characteristic findings on imaging examinations. We searched PubMed for reports of DGCT that included imaging findings written in English beginning in 2017, the time point at which DGCT began to be continuously classified as a benign odontogenic tumor. Fifteen reports describing 16 cases of DGCT with imaging findings were reviewed [1, 3, 7–19] (Table 1). The patients’ ages ranged from 11 to 80 years. Six cases occurred in the maxilla [3, 7, 8, 12, 13, 18], and 10 occurred in the mandible [1, 9–11, 13–17, 19]. Panoramic images were obtained in 12 cases [1, 3, 8–11, 13, 14, 16, 17, 19], a Waters image was obtained in 1 case [18], and CT images were obtained in 11 cases [1, 3, 7–9, 11–14, 17, 19].

**Table 1:** List of reports of dentinogenic ghost cell tumor with image findings written in English from 2017

Authors and years	Cases	Age	Sex	Site	Panoramic image findings	Waters image findings	CT findings	MRI findings
Okui et al. (2023) [7]	1	32	F	Maxilla	NA	NA	Well-defined, low density	NA
Toyodome et al. 2023 [3]	1	60	M	Maxilla	Multilocular, radiolucent	NA	Well-defined, multilocular, low density, high-density structures	NA
Hammad et al. (2023) [1]	1	48	F	Mandible	Well-defined, multilocular, radiolucent	NA	Well-defined, multilocular, low density	NA
Alzaid et al. (2022) [8]	1	42	M	Maxilla	Well-defined, radiolucent	NA	Well-defined, low density	NA
Urs et al. 2022 [9]	1	17	M	Mandible	Well-defined, multilocular, radiolucent	NA	Well-defined, multilocular, low density	NA
Reddy et al. 2022 [10]	1	57	F	Mandible	Multilocular, radiolucent	NA	NA	NA
Novembre et al. (2021) [11]	1	60	M	Mandible	Radiolucent	NA	Low density, high-density structures	NA
Salgado et al. (2021) [12]	1	47	F	Maxilla	NA	NA	Well-defined, multilocular, low density	NA
Bavle et al. (2020) [13]	2	28	M	Maxilla	Well-defined, multilocular, radiolucent, radiopaque calcification	NA	Well-defined, multilocular, low density, high-density structures	NA
		21	F	Mandible	Well-defined, multilocular, radiolucent, radiopaque calcification	NA	NA	NA
Natani et al. (2020) [14]	1	11	M	Mandible	Well-defined, multilocular, radiolucent	NA	NA	NA
Patankar et al. (2019) [15]	1	18	M	Mandible	NA	NA	Well-defined, multilocular, low density, high-density structures	NA
Bussari et al. (2019) [16]	1	40	F	Mandible	Multilocular, radiolucent, radiopaque calcification	NA	NA	NA
Agrawal et al. (2017) [17]	1	14	M	Mandible	Radiolucent, radiopaque calcification	NA	Multilocular, low density, high-density structures	NA
Walia et al. (2017) [18]	1	80	M	Maxilla	NA	Radiolucent, radiopaque calcification	NA	NA
Sheikh et al. (2017) [19]	1	65	F	Mandible	Radiolucent, radiopaque calcification	NA	Low density, high-density structures	NA

The panoramic images and the Waters image in these previous reports showed a well-defined uni- or multilocular radiolucent region, radiopaque calcification, and root resorption [1, 3, 8–11, 13, 14, 16–19]. In our case, the panoramic image showed a relatively well-defined and unilocular radiolucent region in the molar area of the right maxilla. The lesion occupied the right maxillary sinus, and root resorption of tooth 16 was suspected. Our findings of a well-defined border, unilocular radiolucent region, and root resorption were consistent with these previous reports. However, tooth displacement and calcification were not observed.

On CT images in these previous reports, the tumor generally appeared as a well-defined, uni- or multilocular, low-density region with high-density structures and cortical bone expansion [1, 3, 7–9, 11–14, 17, 19]. In our case, CT images showed a well-defined, multilocular low-density region with cortical bone expansion, consistent with previous reports. However, although we had suspected that the minute high-density region around the molar teeth represented alveolar bone change, it instead represented dentin formation. This resulted in difficultly diagnosing the lesion.

MRI was subsequently performed, the mass showed homogeneous isointensity on T1WI, and heterogeneous hypointensity to hyperintensity around the alveolar region but most of the mass showed homogeneous strong hyperintensity on STIR images. In addition, heterogeneous enhancement was observed around the alveolar region at the lower half site of the lesion, and enhancement was present along the edge of the upper region on contrast-enhanced T1WI. We judged that the mass was divided into a solid region and cystic region. The ADC was at 1.5 × 10^−3^ mm^2^/s around the alveolar region at the lower site of the lesion, and high at 2.8 × 10^−3^ mm^2^/s at the upper site of the lesion. The CI curve rapidly increased and reached a plateau at approximately 30 s, and the plateau was sustained to approximately 400 s. We previously reported the CI curves of ameloblastoma calculated from dynamic contrast-enhanced MRI parameters [20–24]. These curves could be divided into two patterns. In one pattern, the curve increased and reached a plateau at 100 to 300 s, and the plateau then either remained unchanged or gradually decreased to 600 to 900 s. In the other pattern, the curve increased relatively rapidly and reached a plateau at 90 to 120 s, decreased relatively rapidly to 300 s, and then decreased gradually thereafter. The CI pattern in the present case was similar to the former pattern, although the plateau was reached earlier. We considered that a benign tumor could be suspected and that a malignant tumor could be differentiated at least from the pattern of the CI curve. To the best of our knowledge, no study to date has focused on the MRI findings of DGCT. Our MRI findings in this case report are the first such findings reported worldwide, and they show the difference in intensity between the solid region and cystic region of the DGCT. Therefore, we believe that this case is extremely valuable and meaningful in a clinical context.

Histopathologically, DGCT is a benign odontogenic tumor consisting of epithelial neoplastic islands that resemble ameloblastoma and are accompanied by ghost cells and dentin [1–3]. According to the WHO, a proportion of ghost cells and dentin exceeding 1% to 2% is useful for the diagnosis of DGCT [2, 5]. In the present case, the tumor comprised epithelial neoplastic islands resembling ameloblastoma inside tight fibroconnective tissue. Masses of ghost cells and formation of dentin were also observed. As a result, the minute high-density area around the molar teeth that we suspected to represent alveolar bone change on the CT images was actually the formation of dentin. These findings were consistent with the typical pathologic findings of DGCT [1–3]. However, at the boundary between the solid area and cystic area, epithelial neoplastic islands resembling ameloblastoma were present whereas ghost cells were absent. The epithelium relining the cystic area was thin stratified squamous epithelium similar to that seen in a dentigerous cyst. To the best of our knowledge, no reports to date have described the pathologic findings of the cystic area of DGCT, making the present findings very valuable.

In conclusion, we have presented a rare case of DGCT that occurred in the maxilla with bone expansion, and we focused particularly on new imaging findings (especially MRI). An accurate imaging diagnosis of DGCT is difficult because of its low frequency and often ambiguous findings, such as the presence or absence of an impacted tooth without obvious calcification. The minute high-density area around the molar teeth that we suspected to be alveolar bone change on the CT images was subsequently found to be the formation of dentin in the histopathologic examination. These findings made it difficult to determine the differential diagnoses of lesions with calcification. The present case suggests that we should consider the possibility of an odontogenic tumor with calcification when high-density structures are observed inside the lesion.

## Data Availability

The data that support the findings of this case report are available from the corresponding author upon reasonable request.

## References

[1] Hammad Y, Bueno S, McLean-Holden A, Schlieve T. Dentinogenic ghost cell tumor: a case report and review of the literature. Oral Maxillofac Surg. 2023;27:169–73. 10.1007/s10006-021-01034-x. 35098400 10.1007/s10006-021-01034-x

[2] de Souza VG, de Pinho MP, Rozza-de-Menezes RE, Cunha KSG, Conde DC. Comparative analysis between dentinogenic ghost cell tumor and ghost cell odontogenic carcinoma: a systematic review. Head Neck Pathol. 2021;15:1265–83. 10.1007/s12105-021-01347-z. 34128137 10.1007/s12105-021-01347-z PMC8633206

[3] Toyodome S, Wakasa T, Hirose K, Iwamoto N, Suzuki S, Nemoto N, et al. Dentinogenic ghost cell tumor treated with a combination of marsupialization and radical resection: a case report and review of the literature. J Med Case Rep. 2023;17:114. 10.1186/s13256-023-03861-w. 36991521 10.1186/s13256-023-03861-w PMC10061976

[4] Carlos R, Ledesma-Montes C. Dentinogenic ghost cell tumour. In: El- Naggar AK, Chan JKC, Grandis JR, Takata T, Slootweg PJ, editors. WHO clas- sification of head and neck tumours. 4th ed. Lyon: IARC; 2017. p. 226–7.

[5] Speight P, Ledesma-Montes C, Wright JM. Calcifying odontogenic cyst. In: El-Naggar AK, Chan JKC, Grandis JR, Takata T, Slootweg PJ, editors. WHO classification of head and neck tumours. 4th ed. Lyon: IARC; 2017. p. 239–41.

[6] Luo HY, Li TJ. Odontogenic tumors: a study of 1309 cases in a Chinese population. Oral Oncol. 2009;45:706–11. 10.1016/j.oraloncology.2008.11.001. 19147397 10.1016/j.oraloncology.2008.11.001

[7] Okui T, Morioka R, Iwahashi T, Matsuda Y, Ishizuka S, Okuma S, et al. A rare case of dentinogenic ghost cell tumor with concomitant odontoma. Clin Case Rep. 2023;11: e7442. 10.1002/ccr3.7442. 37305890 10.1002/ccr3.7442 PMC10248198

[8] Alzaid MA, Kavarodi AM, AlQahtani WM, AlJanobi HA. Recurrent dentinogenic ghost cell tumor: a case report. Am J Case Rep. 2022;23:e936787. 10.12659/AJCR.936787. 35996339 10.12659/AJCR.936787 PMC9427123

[9] Urs AB, Jot K, Maheswari R, Gupta A, Mohanty S. Dentinogenic ghost cell tumor associated with odontoma: a unique histopathological entity and its surgical management. J Clin Pediatr Dent. 2022;46:148–51. 10.17796/1053-4625-46.2.10. 35533231 10.17796/1053-4625-46.2.10

[10] Reddy V, Wadhwan V, Singh R, Bansal V. Dentinogenic ghost cell tumor: case report of a rare central variant and literature review. J Oral Maxillofac Pathol. 2022;26:S68–72. 10.4103/jomfp.jomfp_174_21. 35450252 10.4103/jomfp.jomfp_174_21 PMC9017846

[11] Novembre D, Giofrè E, Barca I, Ferragina F, Cristofaro MG. A rare case of mandibular dentinogenic ghost cell tumor: Histopathological, clinical and surgical management. J Oral Maxillofac Pathol. 2021;25:206. 10.4103/jomfp.JOMFP_185_20. 34349449 10.4103/jomfp.JOMFP_185_20 PMC8272492

[12] Salgado I, Vilares M, Nogueira R, Rito M, Rosa F, Gomes P. Dentinogenic ghost cell tumor—case report of a rare entity. Int J Surg Case Rep. 2021;81: 105651. 10.1016/j.ijscr.2021.105651. 33773371 10.1016/j.ijscr.2021.105651 PMC8024660

[13] Bavle RM, Muniswamappa S, Makarla S, Venugopal R. Variations in aggressive and indolent behaviour of central dentinogenic ghost cell tumor. Case Rep Dent. 2020;2020:8837507. 10.1155/2020/8837507. 33224534 10.1155/2020/8837507 PMC7673946

[14] Natani A, Borah S, Borah M, Agarwal S, Bajpai M. Dentinogenic ghost cell tumor of mandible in a pediatric patient with dysplastic changes. Int J Clin Pediatr Dent. 2020;13:S119–21. 10.5005/jp-journals-10005-1884. 34434027 10.5005/jp-journals-10005-1884 PMC8359891

[15] Patankar SR, Khetan P, Choudhari SK, Suryavanshi H. Dentinogenic ghost cell tumor: a case report. World J Clin Oncol. 2019;10:192–200. 10.5306/wjco.v10.i4.192. 31114751 10.5306/wjco.v10.i4.192 PMC6506423

[16] Bussari S, Thakur SM, Koshy AV, Shah AA. Dentinogenic ghost cell tumor—a case report and review of literature. J Oral Maxillofac Pathol. 2019;23:66–8. 10.4103/jomfp.JOMFP_123_18. 30967728 10.4103/jomfp.JOMFP_123_18 PMC6421922

[17] Agrawal Y, Naidu GS, Makkad RS, Nagi R, Jain S, Gadewar DR, et al. Dentinogenic ghost cell tumor-a rare case report with review of literature. Quant Imaging Med Surg. 2017;7:598–604. 10.21037/qims.2017.03.06. 29184770 10.21037/qims.2017.03.06 PMC5682399

[18] Walia C, Kashyap B, Roy S. Disorganized histomorphology: dentinogenic ghost cell tumor. J Oral Maxillofac Pathol. 2017;21:154–7. 10.4103/jomfp.JOMFP_95_15. 28479706 10.4103/jomfp.JOMFP_95_15 PMC5406799

[19] Sheikh J, Cohen MD, Ramer N, Payami A. Ghost cell tumors. J Oral Maxillofac Surg. 2017;75:750–8. 10.1016/j.joms.2016.10.013. 27865804 10.1016/j.joms.2016.10.013

[20] Asaumi J, Matsuzaki H, Hisatomi M, Konouchi H, Shigehara H, Kishi K. Application of dynamic MRI to differentiating odontogenic myxomas from ameloblastomas. Eur J Radiol. 2002;43:37–41. 10.1016/s0720-048x(01)00453-3. 12065119 10.1016/s0720-048x(01)00453-3

[21] Asaumi J, Hisatomi M, Yanagi Y, Matsuzaki H, Choi YS, Kawai N, et al. Assessment of ameloblastomas using MRI and dynamic contrast-enhanced MRI. Eur J Radiol. 2005;56:25–30. 10.1016/j.ejrad.2005.01.006. 16168260 10.1016/j.ejrad.2005.01.006

[22] Hisatomi M, Yanagi Y, Konouchi H, Matsuzaki H, Takenobu T, Unetsubo T, et al. Diagnostic value of dynamic contrast-enhanced MRI for unilocular cystic-type ameloblastomas with homogeneously bright high signal intensity on T2-weighted or STIR MR images. Oral Oncol. 2011;47:147–52. 10.1016/j.oraloncology.2010.11.009. 21168358 10.1016/j.oraloncology.2010.11.009

[23] Hara M, Matsuzaki H, Katase N, Yanagi Y, Unetsubo T, Asaumi J, et al. Central odontogenic fibroma of the jawbone: 2 case reports describing its imaging features and an analysis of its DCE-MRI findings. Oral Surg Oral Med Oral Pathol Oral Radiol. 2012;113:e51–8. 10.1016/j.oooo.2011.12.013. 22668718 10.1016/j.oooo.2011.12.013

[24] Fujita M, Matsuzaki H, Yanagi Y, Hara M, Katase N, Hisatomi M, et al. Diagnostic value of MRI for odontogenic tumours. Dentomaxillofac Radiol. 2013;42:20120265. 10.1259/dmfr.20120265. 23468124 10.1259/dmfr.20120265 PMC3635777

